# CT-based radiomics modeling for skull dysmorphology severity and surgical outcome prediction in children with isolated sagittal synostosis: a hypothesis-generating study

**DOI:** 10.1007/s11547-022-01493-6

**Published:** 2022-05-10

**Authors:** Rosalinda Calandrelli, Luca Boldrini, Huong Elena Tran, Vincenzo Quinci, Luca Massimi, Fabio Pilato, Jacopo Lenkowicz, Claudio Votta, Cesare Colosimo

**Affiliations:** 1 Department of Diagnostic Imaging, Oncological Radiotherapy, and Hematology, UOC Neuroradiology, Fondazione Policlinico Universitario Agostino Gemelli IRCCS, Rome, Italy; 2 Department of Diagnostic Imaging, Oncological Radiotherapy, and Hematology, Fondazione Policlinico Universitario Agostino Gemelli IRCCS, Rome, Italy; 3 Pediatric Neurosurgery, Neurosurgery Department, Fondazione Policlinico Universitario Agostino Gemelli IRCCS, Largo A. Gemelli, 1, 00168 Rome, Italy; 4 Università Cattolica del Sacro Cuore, Rome, Italy; 5 Unit of Neurology, Neurophysiology, Neurobiology, Department of Medicine, Campus Bio-Medico University, Rome, Italy

**Keywords:** High-resolution CT, Sagittal synostosis, Scaphocephalic severity, Radiomics, Predictive model

## Abstract

**Purpose:**

To investigate the potentialities of radiomic analysis and develop radiomic models to predict the skull dysmorphology severity and post-surgical outcome in children with isolated sagittal synostosis (ISS).

**Materials and methods:**

Preoperative high-resolution CT scans of infants with ISS treated with surgical correction were retrospectively reviewed. The sagittal suture (ROI_entire) and its sections (ROI_anterior/central/posterior) were segmented. Radiomic features extracted from ROI_entire were correlated to the scaphocephalic severity, while radiomic features extracted from ROI_anterior/central/posterior were correlated to the post-surgical outcome. Logistic regression models were built from selected radiomic features and validated to predict the scaphocephalic severity and post-surgical outcome.

**Results:**

A total of 105 patients were enrolled in this study. The kurtosis was obtained from the feature selection process for both scaphocephalic severity and post-surgical outcome prediction. The model predicting the scaphocephalic severity had an area under the curve (AUC) of the receiver operating characteristic of 0.71 and a positive predictive value of 0.83 for the testing set. The model built for the post-surgical outcome showed an AUC (95% CI) of 0.75 (0.61;0.88) and a negative predictive value (95% CI) of 0.95 (0.84;0.99).

**Conclusion:**

Our results suggest that radiomics could be useful in quantifying tissue microarchitecture along the mid-suture space and potentially provide relevant biological information about the sutural ossification processes to predict the onset of skull deformities and stratify post-surgical outcome.

## Introduction

The sagittal suture closure normally begins around 20 years of age and most individuals have it closed between 30 and 50 years old [1]. The premature closure of the sagittal suture is the most common form of isolated suture synostosis (ISS), with an incidence of approximately 1 in 5 000 and a 4:1 male-to-female ratio [2].

The diagnostic phenotype in ISS is characterized by antero-posteriorly expanded neurocranium (scaphocephaly or dolichocephaly), variable bony ridging over the sagittal suture, frontal bossing, occipital protuberance, biparietal and bitemporal narrowing [2, 3].

The diagnosis of ISS is generally established on the basis of clinical observation alone. However, the introduction of high-resolution CT (HR-CT) with three-dimensional reconstructions (3D-CT) demonstrated that the sagittal suture may show a variable degree of synostosis (partially or completely fused) and the synostotic process may progress over time along the sagittal suture toward the skull base [1, 4]. Thus, HR-CT may be useful to early identify the degree of premature synostosis of major and minor sutures of the sagittal ring and to exclude the synostotic involvement of other sutural arches [1].

Patients with ISS may be stratified in different subtypes according to the timing and initial site of sagittal synostosis, and to the over-growth compensatory direction along adjacent major and minor skull sutures [5]. The site of initial synostosis (anterior, posterior, central) is not always related to the length of sagittal synostosis [6]; this is confirmed by volumetric studies that did not find a univocal correlation between the skull volume and the length of sagittal synostosis [7].

The compensatory skull growth, occurring not only at the sutures lying in close proximity to the synostosed sagittal suture (coronal and lambdoidal sutures), but also at minor sutures at the skull base (e.g., sphenopetrosal, sphenosquamosal and sphenofrontal sutures), contributes to influence the overall severity of the skull deformity [8, 9].

The cephalic index assesses the altered length–width ratio and represents a robust and validated tool to quantify the severity of sagittal scaphocephaly in order to plan a patient-tailored surgical approach (i.e., timing of surgery and most appropriate technique) and achieve optimal cosmetic results.

Although the cephalic index indirectly reflects the ossification processes along the synostotic suture and the compensatory over-growth along the patent sutures, its values depend on the plane used for the measurements [10] and may be influenced by the individual conformation of the skull.

The quantification of the ossification degree along the synostotic sagittal suture could therefore theoretically support clinicians in determining the scaphocephalic severity with greater accuracy.

Radiomics represents an innovative development of bio-imaging quantitative analysis based on features extraction from standard radiological images and may provide new insights on skull dysmorphology, integrating the traditional clinical and radiological evaluation [11].

Although radiomics has already been widely applied in oncology, its application in the pediatric imaging field is still scarce [12] and no study has been performed about sutural synostosis, with the aim to evaluate skull shape severity.

It is therefore likely to assume that radiomic features extracted from the sagittal suture from HR-CT images might reflect bone structure changes along the suture synostosis, quantitatively describing the timing of ossification processes and the microstructural bone changes, potentially better correlating with scaphocephaly severity if compared to standard practice.

Aim of this study was to investigate the potentialities of radiomic analysis and develop a HR-CT radiomics-based model able to predict skull dysmorphology severity in children affected by ISS. Additionally, potential radiomic predictors for post-surgical outcomes have been investigated, in order to provide physicians with fully personalized advanced clinical decision support systems.

The main endpoint was to extract radiomic features from the sagittal suture and its sections able to evaluate the most severe scaphocephalic deformities and the poorest post-surgical outcomes.

## Materials and methods

### Patients

We retrospectively reviewed preoperative multiplanar high-resolution 3D-CT images of 105 infants with ISS consecutively admitted to our hospital between 2015 and 2019 (28 females and 77 males; mean age 121 ± 28 days, range 90–180 days). All patients underwent a phosphor-calcic biology test to rule out metabolic disorders and bone deformities (none of them showed alterations).

All infants were characterized by scaphocephaly, variable bony ridging over the sagittal suture, frontal bossing and occipital protuberance.

All infants underwent surgical correction of sagittal craniosynostosis within 2–5 months of life.

The study was approved by the local Institutional Review Board.

### Imaging

HR-CT scans were performed using a GE Light Speed Pro 64 system (GE Medical System, Milwaukee, WI, USA) equipped with an automatic tube current modulation technique (“AutomA”) for low-dose scanning.

Scans were acquired with a 1.25- or 0.6-mm axial slice thickness and data were processed using bone algorithms in addition to 2D-CT and 3D-CT reconstructions.

Scanning was performed under spontaneous sleeping or sedation conditions for all patients to avoid movements.

HR-CT was used to assess the status of the sagittal suture and to exclude the involvement of the other sutures along the sagittal arch and remaining “sutural arches.” The sagittal suture was subdivided into three sections based on their anatomical location (anterior–central–posterior) and each section was scored as “fused” (*f*) or “patent” (*p*) (Table 1).

**Table 1:** Classification of the patients based on the cephalic index and synostotic status of each section of the sagittal suture (anterior–central–posterior sections)

	Classification of patients	
	Dolichocephalic skull morphology	Hyperdolichocephalic skull morphology
Cephalic index	77–66%	< 66%
Patients (*n*.)	26	79

	Segments of sagittal synostosis					
	fff	pff	ffp	ppf	pfp	fpp
Patients (*n*.)	40	25	27	1	11	1

*f* fused, *p* patent, *n* number of patients

#### Skull shape evaluation by cephalic index and surgical outcomes after craniosynostosis reconstruction

**Fig. 1:**
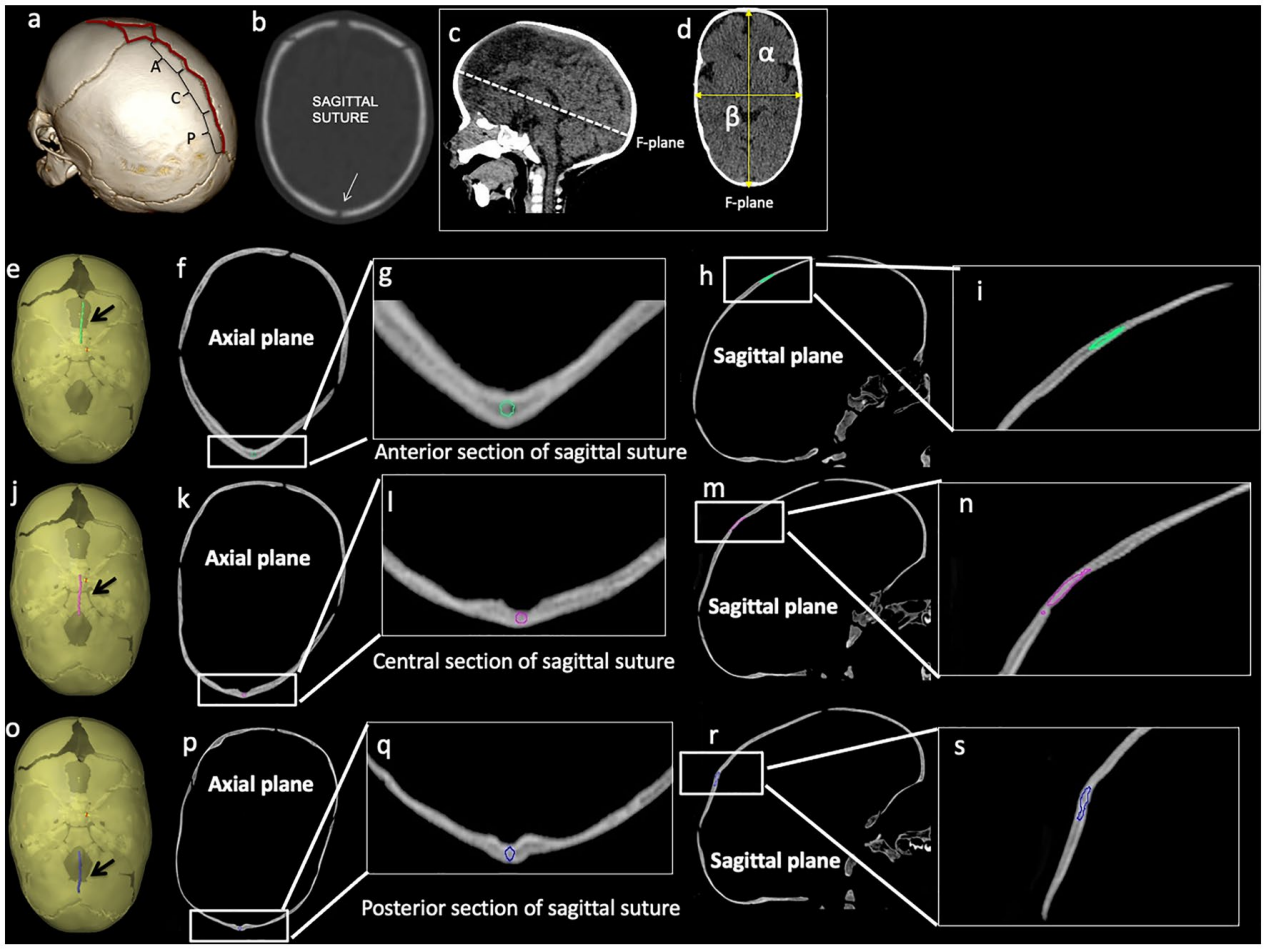
Sagittal suture and skull shape severity quantification using cephalic index. 3D (**a**) and 2D-CT (**b**–**d**). Sagittal suture was subdivided into three sections based on anatomical location (anterior–central–posterior). Patent sagittal suture in b: white arrow. Cephalic index (**c**, **d**) represents the ratio of maximum cranial width to maximum cranial length × 100. F plane: the plane at the level of foramina of Monro was used for measurements. Segmentation of sagittal suture on CT images for radiomic features extraction. 3D (**e**–**j**–**o**) and 2D-CT (**f**, **g**, **h**, **k**, **l**, **m**, **n**, **p**, **q**, **r**, **s**). Anterior (ROI_anterior: green in **e**, **f**, **g**, **h**, **i**; arrow in **e**), central (ROI_central: pink in **j**, **k**, **l**, **m**, **n**; arrow in **j**) and posterior (ROI_posterior: purple in **o**, **p**, **q**, **r**, **s**; arrow in **o**) section of the sagittal suture. The segmentation of the entire sagittal suture (ROI_entire) is the union of the anterior, central and posterior sections

HR-CT images were used to compute the cephalic index. The cephalic index (CeI) represents the ratio of maximum cranial width to maximum cranial length × 100, performed on the F plane (Fig. 1).

We subdivided our patients in two subgroups according to the previously published data about CeI: hyperdolichocephalic (CeI < 66%) and dolichocephalic (CeI in the range 66–77%) skull morphology [9, 10] (Table 1).

The cosmetic results after surgical intervention were evaluated according to the outcome classification proposed by Sloan et al. In this system, classes I to IV represent “excellent” to “good” overall correction of the deformity [13].

### Image postprocessing and radiomic feature extraction

The HR-CT images were manually segmented by a skilled pediatric radiologist using a dedicated image segmentation software (Eclipse, Varian Medical Systems, CA, USA). The segmentations were then independently reviewed by a senior pediatric radiologist as an independent quality check.

The following regions of interest (ROIs) were segmented for each patient: the entire sagittal suture (ROI_entire); the anterior (ROI_anterior), central (ROI_central) and posterior (ROI_posterior) sections of the sagittal suture, according to the subdivision performed for the analysis of the sutural pattern (Fig. 1).

Then, the images in dicom format and the corresponding segmentations were processed with MODDICOM, an open-source R library developed by the Radiomics facility of our institution [14] fully compliant with the Image Biomarker Standardization Initiative recommendations [15]. 

**Fig. 2:**
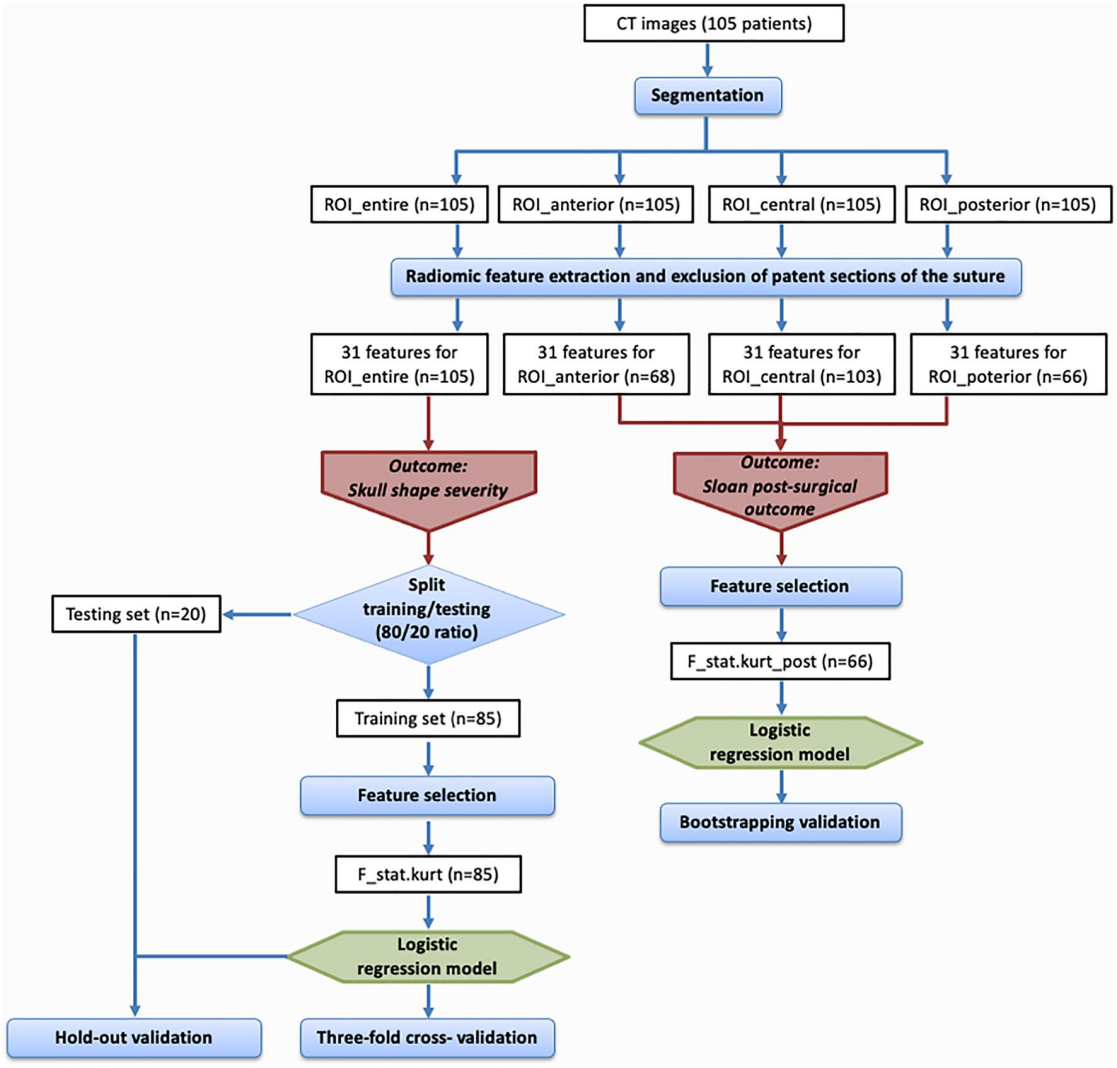
Workflow Diagram. CT images were segmented in different regions of interest (ROIs). The radiomic features extracted from the entire sagittal suture (ROI_entire) were correlated to the skull shape severity. The dataset was split into training (80%) and testing (20%) sets. The training set was used for feature selection, model training and cross-validation. The testing set was used for hold-out validation. The features extracted from anterior (ROI_anterior), central (ROI_central) and posterior (ROI_posterior) sections of the sagittal suture were correlated to the post-surgical outcome. A logistic regression model was trained on a selected feature and validated via bootstrapping

Radiomic features were extracted from each ROI and normalized with their maximum values. For each ROI, 17 intensity-based statistical features described the properties of the gray-level histogram and 14 morphological features described the geometric properties (Fig. 2). Textural radiomic features describing the local distribution of the image gray levels were not included in this study because they were considered not meaningful due to the small size of the ROIs.

### Radiomics study design

#### Skull dysmorphology severity prediction

We firstly developed and validated a radiomic model to predict the skull dysmorphology severity for decision support, based on the features extracted from “ROI_entire.”

We considered the two classes of skull dysmorphology severity based on the CeI: class 0 comprising the dolichocephalic subgroup and class 1 comprising the hyperdolichocephalic subgroup.

The total cohort was then randomly subdivided into a training and a testing dataset, resulting in a training/testing ratio of approximately 80/20 (Table 2).

**Table 2:** Subdivision of patients and fused sections of the suture for the binary classifications

	Skull shape severity	
	Class 0 (dolichocephalic skull morphology) (*n*.)	Class 1 (hyperdolichocephalic skull morphology) (*n*.)
Training set	21	64
Testing set	5	15

	Sloan post-surgical outcome	
	Class 0 (Sloan I) (n.)	Class 1 (Sloan II, III, IV) (n.)
ROI_anterior	54	14
ROI_central	85	18
ROI_posterior	55	11

*n* number of patients

The training dataset was used for radiomic feature selection and model training with internal validation. The testing dataset was used to validate the model with a “held-out” dataset assessing the generalizability of the model with previously unseen data.

#### Post-surgical outcome prediction

The radiomic features extracted from the pathological fused sections of the suture were considered as potential predictors to differentiate between different Sloan post-surgical outcomes. The analysis was conducted for each section location separately.

In order to enhance the clinical translational value of the model, we dichotomized the Sloan post-surgical outcomes into two classes according to the excellent versus good/modest overall correction of the skull deformity: class 0 comprising the Sloan outcome I and class 1 comprising Sloan outcomes II, III and IV (Table 2; Fig. 2).

We hypothesized that the features of the fused sections of the suture (ROI_anterior/central/posterior) may be able to differentiate between excellent (class 0) and good/modest (class 1) post-surgical outcomes.

### Statistical analysis

Statistical analysis and modeling were performed in RStudio (R version 3.4.1).

#### Distribution of the fused sections and scaphocephalic severity

The Fisher statistical test was used to evaluate firstly if the number and position of the fused sections of the sagittal suture were associated with the two classes of skull shape severity, and secondly if the scaphocephalic severity based on the CeI was associated with the two classes of Sloan outcome.

#### Radiomics modeling

A univariate analysis was performed for the training dataset to evaluate the association between each radiomic feature extracted from ROI_entire and the binary skull dysmorphology severity via the Wilcoxon–Mann–Whitney statistical test.

Significance level was set at 0.05 and the significant features were used to build logistic regression models.

We selected the best fitting model according to Akaike information criteria (AIC) by stepwise variable selection. AIC aims to compromise between a good fit to the data and the model complexity to prevent overfitting [16]. The 95% confidence interval (CI) for the model coefficients was calculated as the Wald interval [17].

We performed a threefold cross-validation repeated five times for internal validation. The model performance was assessed in terms of area under the curve (AUC) of the receiver operating characteristic (ROC) curve. We set a threshold based on the prevalence of the outcome to compute accuracy, sensitivity and specificity for each repetition. The predictive value for the majority class 1, namely the positive predictive value (PPV), was also calculated. Then, we computed mean and standard deviation values of the metrics over the five repetitions.

Finally, we applied the developed radiomic model to the testing dataset. This “hold-out” validation was assessed by computing the AUC of the ROC curve, accuracy, sensitivity, specificity and PPV.

A separate radiomic model was then set up to predict post-surgical outcomes. Due to the low number of fused sections in class 1 of the dichotomized post-surgical outcome, the analyses were conducted for each location using the whole available dataset for feature selection and model training and only internal validation was performed.

For feature selection, we performed a univariate analysis with the Wilcoxon–Mann–Whitney statistical test.

We set the significance level at 0.05 with the aim to select only statistically significant features and build a logistic regression model. The model performance was evaluated by computing the AUC of the ROC curve and applying 2000 bootstrap resampling to obtain the 95% CI for the AUC.

For the internal model validation, the bootstrapping of the regression coefficients was performed with 1000 bootstrap resampling to calculate the average coefficients and the 95% CI using the bootstrap percentile method.

A threshold based on the prevalence of the outcome was set to compute the sensitivity, specificity and the predictive value for the majority class 0, namely the Negative Predictive Value (NPV), for the model with the average coefficients. The 95% CI for the performance metrics was obtained with the Jeffrey’s method recommended for small sample sizes [17] (Fig. 2).

## Results

**Fig. 3:**
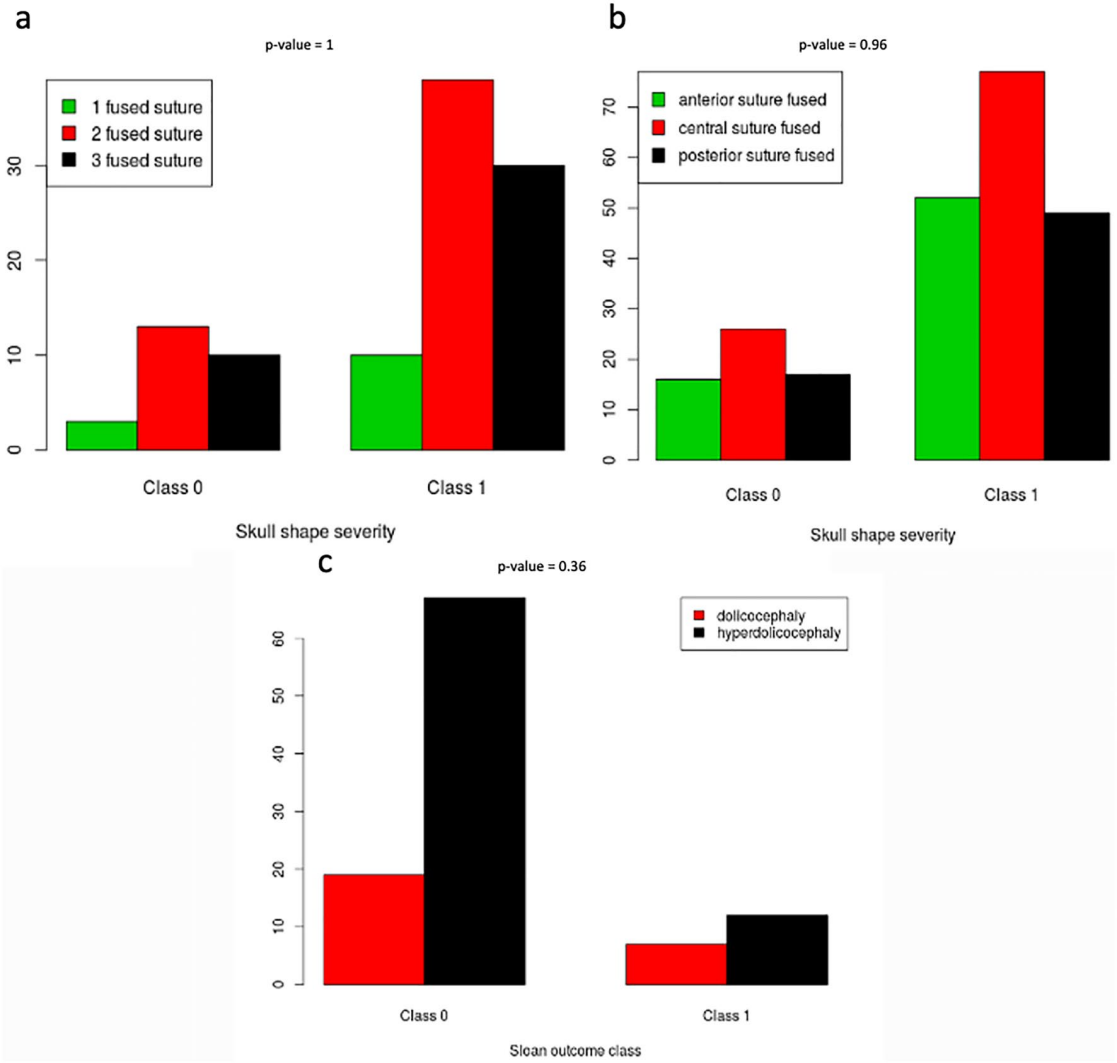
Distribution of the fused sections for the dichotomized scaphocephalic severity. **a**, **b** Barplots indicating the number (**a**) and position (**b**) of the fused sections of the suture for the two classes of skull shape severity (class 0 dolichocephaly, class 1 hyperdolichocephaly). Distribution of the scaphocephalic severity for the dichotomized Sloan outcome. **c** Barplots indicating the clinical severity of the head shape (dolichocephaly, hyperdolichocephaly) for the two classes of Sloan post-surgical outcome (class 0 excellent, class 1 good/modest)

No statistically significant difference between the two classes of skull shape severity was found for the number (*p* value = 1) or for the position (*p* value = 0.96) of the fused sections (Fig. 3a–b).

Similarly, no statistically significant association was found between the clinical severity based on the CeI and the binary post-surgical outcome (*p* value = 0.36) (Fig. 3c).

From the univariate analysis, we found that the kurtosis (F_stat.kurt) and the gray-level histogram 10th percentile showed a statistically significant difference for the two classes of skull dysmorphology severity of *p* = 0.011 and *p* = 0.044, respectively.

The best fitting logistic model according to the AIC included F_stat.kurt as only predictor. The model coefficient estimates (95% CI) were as follows: 1.38 (0.80; 1.96) for the Intercept (*p* value < 0.001) and − 1.94 (− 3.60; − 0.27) for F_stat.kurt (*p* value = 0.022). From the internal validation, the following mean (std) values were obtained: AUC 0.68 (0.07), accuracy 0.71 (0.04), sensitivity 0.72 (0.07), specificity 0.69 (0.23), PPV 0.88 (0.09) (Table 3). The PPV obtained for the logistic model was high when compared to the null model, which was based on the prevalence of the majority class 1 (~ 0.75).

**Table 3:** Coefficients of the logistic regression model based on the entire sagittal suture (ROI_entire) for the prediction of the skull shape severity

Model coefficients		
	Estimate (95% CI)	*p* value
Intercept	1.38 (0.80;1.96)	< 0.001
F_stat.kurt	− 1.94 (− 3.60; − 0.27)	0.022

Model performance			
	Sensitivity	Specificity	PPV
Threefold cross-validation	0.72	0.69	0.88
“Hold-out” validation	0.67	0.60	0.83

Model performance results for the threefold cross-validation and “hold-out” validation

*CI* confidence interval, *PPV* positive predictive value, *F_stat.kurt* kurtosis extracted from the entire sagittal suture

**Fig. 4:**
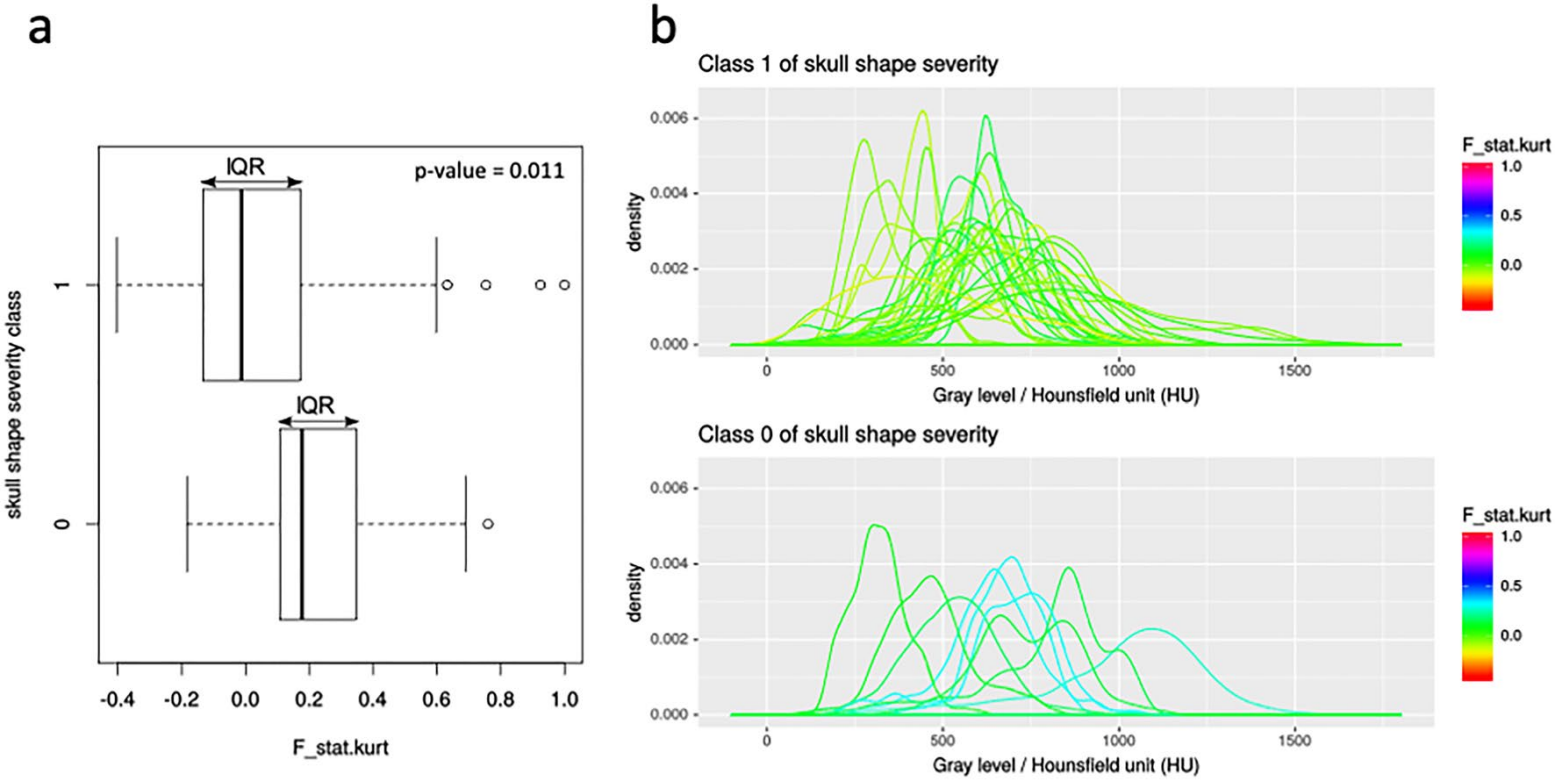
Boxplots (**a**) and density plots (**b**) of the dichotomized skull shape severity. **a** Boxplots of the feature F_stat.kurt extracted from ROI_entire for the two classes of skull shape severity; **b** distributions of gray levels (density plots) within ROI_entire for patients belonging to two classes of skull shape severity and characterized by F_stat.kurt values within the interquartile range (IQR)

The distributions of gray levels with a F_stat.kurt value within the interquartile range (IQR) were mainly centered above 350–500 Hounsfield Units (HU), namely in the compact bone range, for the class 1 of skull shape severity, while they were spread over a wide range of HU, including both soft tissues and bones, for the class 0 of skull shape severity (Fig. 4).

From the application of the proposed model to the testing dataset we obtained performances close to the one yielded in the internal validation. In particular, AUC was equal to 0.71 in the testing dataset, while accuracy, sensitivity, specificity and PPV were 0.65, 0.67, 0.60 and 0.83, respectively (Table 3).

From the univariate analysis, the only statistically significant feature able to discriminate the Sloan post-surgical outcome was the kurtosis extracted from ROI_posterior (*p* value = 0.009) and indicated as F_stat.kurt_post.

**Fig. 5:**
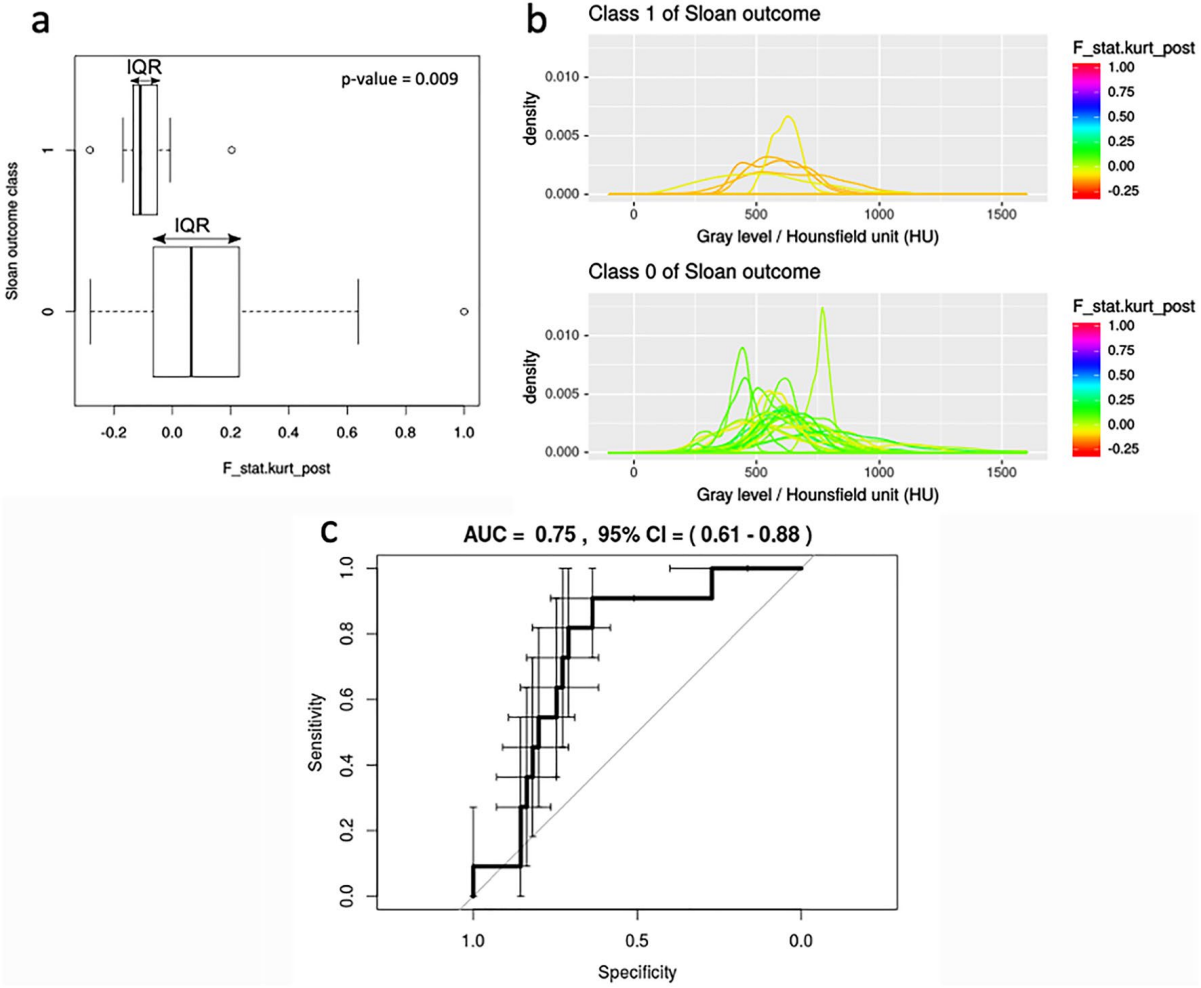
Boxplots (**a**) and density plots (**b**) of the dichotomized Sloan post-surgical outcome for ROI_posterior. **a** Boxplots of the feature F_stat.kurt_post extracted from ROI_posterior for the two classes of Sloan outcome, **b** distributions of gray levels (density plots) within ROI_posterior for patients belonging to the classes 1 and 0 of Sloan outcome and characterized by F_stat.kurt_post values within the interquartile range (IQR). ROC curve of the logistic regression model built for ROI_posterior (**c**). 95% confidence intervals (CI) for the sensitivity and specificity are represented as bars

The distributions of gray levels with a F_stat.kurt_post value within the IQR spread over a wide range of HU, mainly including bones ranges, for both Sloan outcome classes 0 and 1 (Fig. 5a, b).

The predictor F_stat.kurt_post showed indeed a statistically significant estimated model coefficient with an AUC (95% CI) of 0.75 (0.61;0.88) (Fig. 5c).

The model coefficient estimates (95% CI) were as follows: − 1.66 (− 2.39; − 0.93) for the Intercept (*p* value < 0.001) and − 4.82 (− 9.18; − 0.45) for F_stat.kurt_post (*p* value = 0.030). From the bootstrapping, we obtained the following average (95% CI) model coefficients: − 1.73 (− 2.58; − 1.09) for the Intercept and − 5.19 (− 9.69; − 1.78) for F_stat.kurt_post. The corresponding model performance metrics (95% CI) were as follows: sensitivity of 0.82 (0.53;0.96), specificity of 0.64 (0.50;0.75), NPV of 0.95 (0.84;0.99) (Table 4). The NPV was high when compared to the null model, which was based on the prevalence of the majority class 0 (~ 0.83).

**Table 4:** Coefficients of the logistic regression model based on the posterior section of the sagittal suture (ROI_posterior) for the prediction of the Sloan post-surgical outcome

Model coefficients			
	Estimate (95% CI)	Estimate p value	Bootstrapped average (95% CI)
Intercept	− 1.66 (− 2.39; − 0.93)	< 0.001	− 1.73 (− 2.58; − 1.09)
F_stat.kurt_post	− 4.82 (− 9.18; − 0.45)	0.030	− 5.19 (− 9.69; − 1.78)

Model performance		
Sensitivity (95% CI)	Specificity (95% CI)	NPV (95% CI)
0.82 (0.53;0.96)	0.64 (0.50;0.75)	0.95 (0.84;0.99)

Model coefficients validated with bootstrapping. Model performance results for classification

*CI* confidence interval, *NPV* negative predictive value, *F_stat.kurt_post* kurtosis extracted from the posterior fused section of the suture

## Discussion

It is well known that a deregulation of the osteogenic processes represents the main pathogenetic mechanism for sutural synostoses. Such deregulation would probably affect the undifferentiated mesenchymal cells occupying the mid-suture space [18]. In normal development process, mesenchymal cells adjacent to the osteogenic fronts would be able to differentiate into osteoblasts and to be incorporated into the growing bone, while mid-suture mesenchymal cells remained undifferentiated but able to contribute to the calvarial bone growth as well [19]. A “hyperactivity” of those undifferentiated mid-suture mesenchymal cells would produce an early ossification of the intramembraneous space with premature closure of the affected suture.

Unfortunately, the qualitative, radiologist-driven, assessment of the degree of suture fusion and synostosis location along the sagittal suture is not sufficient to thoroughly describe the severity of scaphocephaly [6]. Our data represented by the number and position of the fused sections of the sagittal suture showed no significant association with the skull shape severity, in accordance with these previous studies. Thus, the severity of the overall skull deformity, influenced by compensatory calvarial remodeling, may mainly depend on the ossification processes along the sagittal synostosis.

We suppose therefore that radiomic features extracted along the sagittal suture from HR-CT images might reflect bone structure changes along the suture synostosis according to the timing of ossification processes, and these quantitative features might successfully predict and correlate with the scaphocephaly severity and the clinical outcome.

In the present study, we developed radiomic models using features extracted from HR-CT images of the entire sagittal suture or its partially fused segments, with the aim to stratify the severity of head deformity and stratify clinical outcomes in children presenting isolated sagittal synostosis.

The first step in our study was to correlate the radiomic features extracted from the sagittal suture to overall skull shape severity. As the timing of sutural synostosis influences the ossification processes along the entire sagittal suture and consequently the overall skull shape severity, radiomic features extracted along the entire sagittal suture may be able to better reflect sutural bone changes and predict the overall skull deformity. We observed that a radiomic feature extracted from the entire sagittal suture, namely the kurtosis, was able to stratify our sample in the two groups of skull shape severity, as described by the CeI (dolichocephaly vs. hyperdolichocephaly).

The logistic regression model based on the kurtosis was indeed able to predict the skull shape severity with a high positive predictive value when compared to the null model.

For the class 1 of the skull shape severity (hyperdolichocephalic group), the distributions of gray levels were mainly centered above 350–500 HU, namely in the compact bone range, while for the class 0 of the skull shape severity (dolichocephalic group) the distributions of gray levels spread over a wide range of HU, including both soft tissues and bones ranges.

Our data suggest that most of the patients belonging to class 1 had an advanced stage of ossification and probably an earlier onset of suture synostosis. This was in accordance with previous studies reporting that the earlier the synostosis takes place, the greater the effect on skull shape, and conversely, the later the synostosis occurs, the less effect on skull shape [7, 20].

On the other hand, the difficulty to obtain any univocal density range along the entire sagittal suture in patients belonging to class 0 does not allow evaluating the effective state of the ossification processes in this group. This is probably related to the low number of cases showing incomplete and variable degrees of bone changes available in our patient cohort.

The second step in our study was to correlate the radiomic features extracted from the fused sections of the sagittal suture with the Sloan post-surgical outcome. As a comparison, we also correlated overall severity of head shape based on the cephalic index with the Sloan outcome, and we found that the clinical severity based on the cephalic index alone was not able to differentiate between excellent and good/modest post-surgical outcomes.

With regards to the quantitative radiomic analysis, no radiomic feature extracted from the entire suture presented a significant association with the binary post-surgical outcome. For this reason, we hypothesized that single fused sections of the sagittal synostosis may independently influence the Sloan outcome. We found that the kurtosis extracted from ROI_posterior (F_stat.kurt_post) was the only predictor able to differentiate between the excellent (class 0) and good/modest (class 1) clinical outcomes, supporting a possible prominent role of the posterior segment of this suture in skull deformity onset. The posterior section of sagittal synostosis is considered among the main variables influencing overall skull shape, but other factors such as the action of the growth forces perpendicular to the synostotic posterior segment of the sagittal suture contribute to determine the posterior skull changes. Their synergic effect might affect the bone architecture and composition of the posterior segment of sagittal suture which may be detected by the different kurtosis values allowing to differentiate the two classes of Sloan clinical outcomes.

The distributions of gray levels were centered over the compact bone range for both classes of Sloan outcome and this data confirmed the complete ossification of the posterior suture section.

Future studies need to confirm the predictive value of the proposed model based on the posterior fused section using larger and independent datasets.

In conclusion, our results suggest that the kurtosis is the only radiomic feature extracted from HR-CT images potentially able to evaluate tissue microarchitecture along the sagittal suture and provide relevant biological information about both the overall severity skull shape and clinical outcomes.

## Conclusion

We developed radiomic models from HR-CT images to predict the skull shape deformity and differentiate the clinical outcome in children with isolated sagittal synostosis. As far as the authors know, this experience represents the first application of radiomics for skull deformities prediction.

Our analysis demonstrated that the kurtosis could be used as the only radiomic feature to quantify tissue microarchitecture along the mid-suture space and potentially provide relevant biological information about the sutural ossification processes in order to predict the skull deformity and differentiate the post-surgical cosmetic results, supporting clinicians in their decision. In particular, the kurtosis extracted from the entire sagittal suture was able to predict the skull shape severity, while the kurtosis extracted along the synostotic posterior segment of the sagittal suture was able to differentiate the clinical outcomes.

Considering the promising results obtained, further validation studies with larger sample size and external datasets are planned to confirm our results, paving the way to the introduction of radiomic analysis in children with skull deformity secondary to premature synostosis of the cranial sutures.
